# Flt3L therapy increases the abundance of Treg-promoting CCR7^+^ cDCs in preclinical cancer models

**DOI:** 10.3389/fimmu.2023.1166180

**Published:** 2023-08-09

**Authors:** Emile J. Clappaert, Daliya Kancheva, Jan Brughmans, Ayla Debraekeleer, Pauline M. R. Bardet, Yvon Elkrim, Dagmar Lacroix, Maida Živalj, Ahmed E.I. Hamouda, Jo A. Van Ginderachter, Sofie Deschoemaeker, Damya Laoui

**Affiliations:** ^1^ Laboratory of Dendritic Cell Biology and Cancer Immunotherapy, VIB Center for Inflammation Research, Brussels, Belgium; ^2^ Laboratory of Cellular and Molecular Immunology, Vrije Universiteit Brussel, Brussels, Belgium; ^3^ Laboratory of Myeloid Cell Immunology, VIB Center for Inflammation Research, Brussels, Belgium

**Keywords:** dendritic cell, immunotherapy, breast cancer, Flt3L, combination therapies, lung cancer

## Abstract

Conventional dendritic cells (cDCs) are at the forefront of activating the immune system to mount an anti-tumor immune response. Flt3L is a cytokine required for DC development that can increase DC abundance in the tumor when administered therapeutically. However, the impact of Flt3L on the phenotype of distinct cDC subsets in the tumor microenvironment is still largely undetermined. Here, using multi-omic single-cell analysis, we show that Flt3L therapy increases all cDC subsets in orthotopic E0771 and TS/A breast cancer and LLC lung cancer models, but this did not result in a reduction of tumor growth in any of the models. Interestingly, a CD81^+^migcDC1 population, likely developing from cDC1, was induced upon Flt3L treatment in E0771 tumors as well as in TS/A breast and LLC lung tumors. This CD81^+^migcDC1 subset is characterized by the expression of both canonical cDC1 markers as well as migratory cDC activation and regulatory markers and displayed a Treg-inducing potential. To shift the cDC phenotype towards a T-cell stimulatory phenotype, CD40 agonist therapy was administered to E0771 tumor-bearing mice in combination with Flt3L. However, while αCD40 reduced tumor growth, Flt3L failed to improve the therapeutic response to αCD40 therapy. Interestingly, Flt3L+αCD40 combination therapy increased the abundance of Treg-promoting CD81^+^migcDC1. Nonetheless, while Treg-depletion and αCD40 therapy were synergistic, the addition of Flt3L to this combination did not result in any added benefit. Overall, these results indicate that merely increasing cDCs in the tumor by Flt3L treatment cannot improve anti-tumor responses and therefore might not be beneficial for the treatment of cancer, though could still be of use to increase cDC numbers for autologous DC-therapy.

## Introduction

For many cancer types chemotherapy is still the standard of care. Chemotherapy has recently been complemented with immune checkpoint inhibitors, but the response rates remain low, indicating the need for novel therapies (1). One strategy that has been suggested to improve anti-tumor immunity is to increase the influx of conventional dendritic cells (cDCs) into the tumor microenvironment (TME) by intervening at the very start of the immunity cycle. Besides kickstarting the immunity cycle locally in the tumor, tumor-infiltrating cDCs could then also serve as starting material for autologous cell therapies (2, 3).

To achieve this goal, Fms-like tyrosine kinase 3 ligand (Flt3L), a cytokine required for cDC and plasmacytoid dendritic cell (pDC) development, could be a valid candidate (4). Indeed, systemic treatment with Flt3L, leading to supraphysiological Flt3L levels, resulted in the expansion of cDCs and pDCs in the TME of mouse melanoma and reduced tumor growth when used in combination therapies (5, 6). While increasing cDCs in the tumor has an undeniable potential, the impact of systemic Flt3L treatment on the intra-tumoral DC phenotype has not yet been described in detail.

In the current study, we present an in-depth multi-omic single-cell analysis of the immune compartment in the murine TNBC model E0771, comparing untreated and Flt3L-treated setting, and confirm several findings in the TS/A breast cancer and Lewis lung cancer (LLC) model. Our results show that Flt3L indeed increases cDC abundance in the TME, but does not reduce tumor growth. Interestingly, Flt3L induces a CD81^+^ migratory cDC1 state that can drive Treg-differentiation. Administration of Flt3L therapy with αCD40 or αCD25 mAb therapies to respectively improve cDC activation or deplete Tregs did, however, not lead to added benefit, while the latter two synergistically reduced E0771 tumor growth.

## Results

### Flt3L therapy increases cDC numbers in the TME, but does not reduce tumor growth

**Figure 1:**
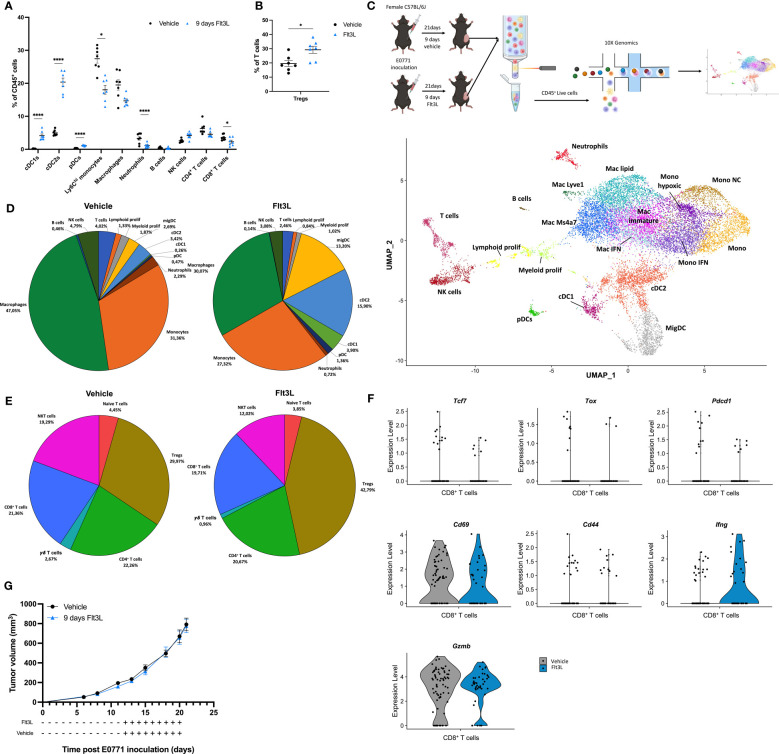
Flt3L treatment significantly increases DCs in the E0771 tumor microenvironment. **(A)** Frequency of immune populations within E0771 tumors treated for 9 days with vehicle or Flt3L. **(B)** Tregs expressed as percentage of T cells of E0771 tumors collected after 9 days of vehicle or Flt3L treatment. **(C)** Graphical representation of the experimental setup and UMAP plot of 9838 cells (vehicle) or 10341 cells (Flt3L) isolated from pools of 3 E0771 tumors at a volume of 789 ± 221 mm^3^ (vehicle) or 773 ± 116 mm^3^ (Flt3L). **(D)** Pie charts representing the frequency of distinct immune populations identified in **(C)**. **(E)** Pie charts of subclustered T-cell subsets from the CITE-seq dataset obtained in **(C)**. **(F)** Violin plots showing the expression of *Tcf7*, *Tox*, *Pdcd1*, *Gzmb*, *Cd69*, *Cd44* and *Ifng* in CD8^+^ T cells identified in **(C)**. **(G)** E0771 tumor growth upon 9 days of vehicle or FLt3L treatment. Representative data from 2 experiments (n=7). *, *P < 0.05*; ****, *P < 0.0001*.

To determine the optimal Flt3L regimen resulting in the highest numbers of cDCs in orthotopic E0771 breast tumors, 30 µg Flt3L or vehicle was administered daily for 6, 9 or 12 days before sacrifice (**Figure S1A**). The highest amount of tumor-cDCs was observed in the 9-day treatment schedule with a 20-fold, 4-fold and 3.6-fold increase in cDC1, cDC2 and pDCs, respectively (**Figures S1B, C**). This regimen was selected for all subsequent experiments. Flt3L treatment also increased cDCs in tumor-draining lymph nodes (tdLN), spleen and bone marrow (**Figures S1D–F**). We hypothesized that the increased numbers of cDCs may result in a higher amount of T cells in the TME. However, the increase in tumor-cDCs within CD45^+^ cells was accompanied by a relative reduction in myeloid cells including monocytes, macrophages and neutrophils, as well as CD8^+^ T cells (**Figure 1A**). Interestingly though, in E0771 tumors, the relative contribution of Tregs to the T-cell compartment significantly increased (**Figure 1B**). To assess the activation state of immune cells upon Flt3L treatment, we performed cellular indexing of transcriptomes and epitopes by sequencing (CITEseq) of CD45^+^ cells sorted from end-stage E0771 tumors, treated with vehicle or Flt3L (**Figure 1C**). Unsupervised clustering of the transcriptomic data resulted in 19 clusters, which were annotated based on canonical marker genes (**Figures 1C**, **S2A**). In line with the flow cytometry data, the percentages of cDC1, cDC2, migratory-DCs [migDCs, also termed mature DCs or mregDCs (7)] and to a lesser extent also pDCs, all increased in the FLT3L-treated tumors (**Figures 1D**, **S2B**). To assess whether the increased amount of cDCs resulted in a stronger activation of T cells, we reanalyzed the T-cell compartment (**Figures 1E**, **S2C, D**). Apart from an increased expression of *Ifng*, the activation state of CD8^+^ T cells remained largely unaltered in tumors containing more cDCs (**Figures 1F**, **S2E**). Along the same line, CD4^+^Foxp3^-^ T cells expressed more *Ifng* and *Pdcd1*, but did not overtly change other activation markers, nor did the Tregs (**Figures S2E, F**). However, the relative abundance of Tregs within the T-cell compartment increased, similar to the prior observation via flow cytometry (**Figure 1E**). Consequently, the tumor growth of E0771 tumor-bearing mice treated with Flt3L was not different from vehicle-treated mice (**Figure 1G**).

### Flt3L therapy induces a CD81^+^migcDC1 activation state

**Figure 2:**
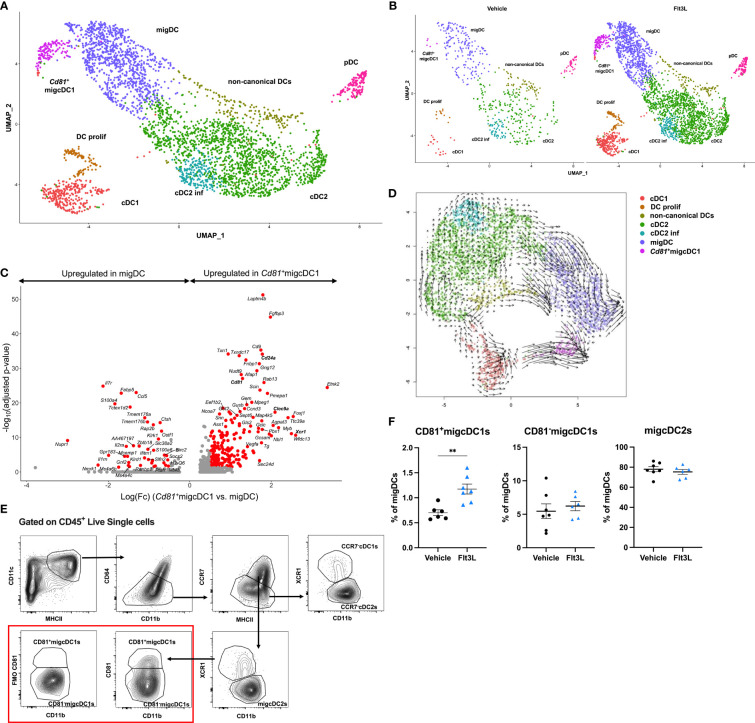
Flt3L therapy induces a CD81^+^migcDC1 activation status. **(A, B)** Merged **(A)** and split **(B)** UMAP plots of subclustered DCs from **Figure 1C**. **(C)** Volcano plot showing genes upregulated in *Cd81* ^+^migcDC1 and migDC with red dots representing significantly upregulated genes. **(D)** Velocity estimates projected onto the UMAP plot of **(B)** The arrows visualize the observed and extrapolated future states of the cells. **(E)** Gating strategy to identify different cDC subsets in E0771 tumors. **(F)** Percentages of CD81^+^migcDC1, CD81^-^migcDC1 and migcDC2 within migDCs. Representative data from 2 independent experiments (n=7). **, *P < 0.01*.

Since Flt3L promotes hematopoietic progenitor commitment to the DC lineage as well as their survival and proliferation (4), one could hypothesize that the Flt3L-induced cDCs arriving in the TME represent a less mature state. However, after 9 days of Flt3L therapy, the expression of DC subset activation markers was not significantly different on DCs infiltrating vehicle-treated *versus* Flt3L-treated tumors, indicating that DCs in Flt3L-treated tumors had acquired a mature state (**Figure S3A**). Interestingly, a reclustering of the DC compartment revealed that Flt3L therapy has not only increased all known DC states, but also prominently induced a distinct DC cluster, characterized by the expression of *Fgfbp3, Laptm4b, Cd81, Scin*, as well as migDC (*Ccr7* and *Cd200*) and cDC1-associated genes (*Xcr1, Clec9a and Cd24a*) (**Figures 2A–C**, **S3B-D**). We therefore annotated this cluster as *Cd81* ^+^migcDC1. RNA velocity analysis further suggested that this *Cd81* ^+^migcDC1 population mainly originated from cDC1s upon Flt3L therapy (**Figure 2D**). The intratumoral presence of CCR7^+^ CD81^+^ migDCs was further confirmed using flow cytometry (**Figure 2E**), and our CITEseq data confirmed the high expression of XCR1 and CD24 at the protein level on these cells (**Figure S3E**). Of note, two additional migDC populations were characterized as CCR7^high^XCR1^+^CD11b^low^CD81^-^ migcDC1s and CCR7^high^XCR1^-^CD11b^int-high^ migcDC2s (**Figure 2E**). Flow cytometry further confirmed that Flt3L therapy increased all DC subsets (**Figure S3F**), but only the CD81^+^migcDC1s increased within the CCR7^+^ DC compartment (**Figure 2F**). Interestingly, these findings could be recapitulated in other preclinical models such as in the TS/A breast cancer model and the Lewis lung carcinoma (LLC) model, where Flt3L therapy also increased the abundance of DCs in the TME (**Figured S4A, B**), but within the CCR7^+^ DC compartment, specifically increased CD81^+^migcDC1s (**Figures S4C–F**). In these models systemic Flt3L therapy also did not lead to a reduction in tumor growth (**Figures S4G, H**).

### Flt3L-induced tumor-associated CD81^+^migcDC1 are potent Treg inducers

**Figure 3:**
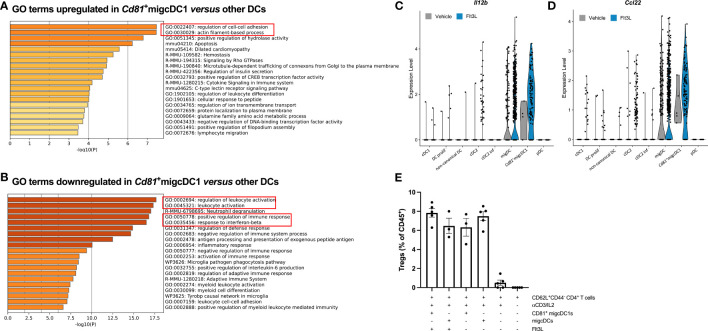
Flt3L-induced tumor-associated CD81^+^migcDC1 are potent Treg inducers. **(A, B)** Gene ontology analysis of differentially upregulated **(A)** or downregulated **(B)** genes in *Cd81* ^+^migcDC1 *versus* all other DCs from **Figure 2A**. **(C, D)** Violin plots of *Il12b* **(C)** and *Ccl22* **(D)** in DC subsets from **Figure 2A**. **(E)** Percentage of Tregs within CD45^+^ cells after coculture of sorted CD81^+^migcDC1 and migDCs (CD81^-^migcDC1 and migcDC2) derived from vehicle or Flt3L-treated animals with naive CD44^-^CD62L^+^CD4^+^T cells. Representative data from 2 independent experiments.

To assess the potential functions of CD81^+^migcDC1, we performed gene ontology (GO) enrichment analysis on the differentially expressed genes in *Cd81* ^+^migcDC1 *versus* all other DC subsets. GO terms related to migration, such as “cell-cell adhesion” and “actin filament-based process”, were enriched for the upregulated genes, highlighting the migratory potential of *Cd81* ^+^migcDC1s (**Figures 3A**, **S5A**). Flt3L-induced CD81^+^migcDC1s indeed expressed the highest levels of CD40, CD80 and PD-L1 compared to other infiltrating DCs in Flt3L-treated tumors (**Figures S5B–D**). Conversely, GO terms such as “leukocyte activation” and “positive influence on the immune response” were downregulated in *Cd81* ^+^migcDC1s, suggesting an immunosuppressive potential (**Figure 3B**). In the first instance, we scrutinized the potential interaction of *Cd81* ^+^migcDC1s with Tregs, as a putative immunosuppressive mechanism. Interestingly, Flt3L-induced *Cd81* ^+^migcDC1 expressed high levels of *Il12b* (**Figures 3C**, **S5A**), a feature that was previously linked with CCR7^+^ DCs that have the potential to interact with Tregs (8). In addition, *Cd81* ^+^migcDC1 also expressed high levels of *Ccl22*, which has been shown to promote the interaction with Tregs (9) and CCL22 was also increased at the protein level in the tumor upon 9 days of Flt3L treatment (**Figures 3D**, **S5A, E**). NicheNet predicted multiple interactions between all tumor-infiltrating DC subsets and Tregs, however, the *Cd81* ^+^migcDC1 showed the strongest evidence of actual interaction (**Figures S5F, G**). Most importantly, when culturing naive T cells with either CD81^+^migcDC1s or other migDCs (composed of CD81^-^migcDC1s and migcDC2s) sorted from Flt3L-treated tumors, the CD81^+^migcDC1s showed a trend to more potently drive the differentiation of naive T cells into Tregs (**Figure 3E**). However, when blocking CCL22 in the cocultures, the number of induced Tregs was not altered, indicating that CCL22 is not the mediator of the Treg-promoting capacity of CD81^+^migcDC1s and other migDCs *in vitro* (**Figure S5H**). Importantly, CD81^+^migcDC1s and other migDCs isolated from vehicle-treated mice could induce Tregs to the same extent as CD81^+^migcDC1s and other migDCs isolated from FLt3L-treated mice (**Figure 3E**). Together, these data demonstrate that Flt3L treatment does not alter the Treg-inducing capacity of CCR7^+^ DCs, but increases the abundance of the DC populations with Treg-inducing capacities.

### Flt3L therapy does not improve the αCD40-mediated reduction of tumor growth

**Figure 4:**
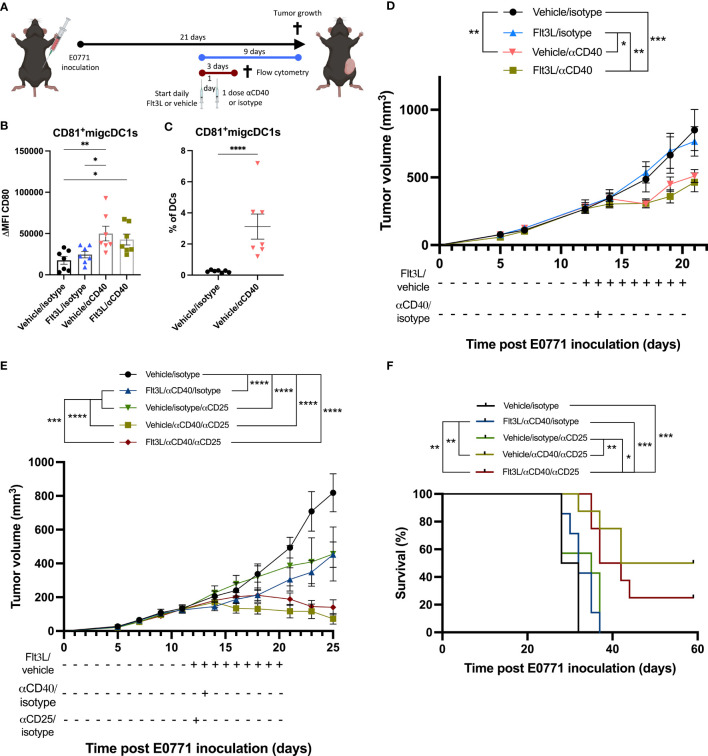
Flt3L therapy does not improve the αCD40-mediated tumor-growth reduction. **(A)** Graphical representation of the experimental design. **(B)** Delta mean fluorescence intensity (ΔMFI) of CD80 in CD81^+^migcDC1 two days after αCD40 as depicted in **(A)**. **(C)** Percentage of CD81^+^migcDC1 within DCs two days after αCD40 as depicted in **(A)**. **(D)** E0771 tumor growth upon Flt3L+αCD40 therapy. **(E, F)** Tumor growth **(E)** and survival **(F)** of E0771 tumor-bearing mice treated with Flt3L +αCD40+αCD25. *, *P < 0.05*; **, *P < 0.01*; ***, *P < 0.001*; ****, *P < 0.0001*.

We next wondered whether CD81^+^migcDC1s could be turned into T-cell stimulating cells. αCD40 agonist therapy has been reported to potently trigger the T-cell activating potential of DCs (10). When analyzing the E0771 TME shortly (48h) after αCD40 therapy, CD81^+^migcDC1s indeed displayed higher levels of CD80 (**Figures 4A, B**). In addition, while the percentage of DCs within tumor-infiltrating CD45^+^ cells was lower in αCD40-treated tumors, the contribution of CD81^+^migcDC1s to the overall DC pool increased (**Figures 4C**, **S6A, B**), suggesting that αCD40 further induced cDC1 to adopt an activated migcDC1 state as has been shown in other tumor models (10). Indeed, CD81^+^migcDC1s were more numerous in Flt3L+αCD40-treated tumors compared to Flt3L-treated tumors (**Figure S6B**). It could then be anticipated that the expansion of cDCs by Flt3L, including CD81^+^migcDC1s, in combination with their activation by αCD40 would result in a better anti-tumor immune response. However, while αCD40 therapy significantly reduced E0771 tumor growth, Flt3L did not provide any additional therapeutic benefit (**Figure 4D**). To understand why Flt3L did not improve the therapeutic effect mediated by αCD40, we assessed the T-cell infiltrate in the TME 48h after αCD40 therapy. While the infiltration of CD8^+^ T cells was not altered, αCD40 therapy decreased the percentages of Tregs (**Figures S6C, D**). However, Tregs partly recovered to normal levels in tumors treated with Flt3L+αCD40 and significantly increased in comparison to αCD40 monotherapy (**Figure S6D**), suggesting that the Flt3L-mediated expansion of the Treg-inducing CD81^+^migcDC1s could be involved in this phenomenon. Notably, CCL22 levels were also increased in the tumor supernatants 48h after αCD40 monotherapy and further increased in αCD40+Flt3L-treated tumors (**Figure S6E**). Based on these findings, we wondered whether depleting Tregs would uncover a putative beneficial effect of Flt3L on anti-tumor immunity. Treg depletion using αCD25 mAb indeed synergized with αCD40 and significantly prolonged E0771 survival, but Flt3L therapy did not further improve the response to αCD25+αCD40 therapy (**Figures 4E, F**). These results suggest that besides Tregs, other factors in E0771 are mediating the lack of therapeutic benefit of Flt3L treatment.

## Discussion

To improve breast cancer therapy, we hypothesized that kickstarting the immunity cycle by increasing tumor DC numbers could be beneficial. Several cytokines have been shown to increase cDC numbers including GM-CSF, Flt3L, XCL1, CCL4, CCL5 or CCL20 (11, 12). Nonetheless, GM-CSF, CCL4, CCL5 and CCL22 have a more pleiotropic effect on not only the myeloid cell compartment, but also for example, epithelial cells, endothelial cells and fibroblasts (13–16). On the other hand, XCL1 is more specific for cDC1 attraction, while both cDC1 and cDC2 have been shown to be relevant for anti-tumor immunity (10, 17). In contrast, Flt3L has been shown to increase both cDC1 and cDC2 and the impact on other cell types is limited to immune cell populations. Indeed, also pDCs, NK cells, B cells, monocytes, red pulp macrophages, granulocytes and innate lymphoid cells have been shown to expand upon Flt3L treatment (18–22). In addition, systemic Flt3L therapy has already been evaluated in patients and has a good safety profile. In these patients, a clear increase in the cDC compartment in the blood is observed and results in an enhanced CD8^+^ T-cell response in response to DC targeting vaccines (23). In addition, a window of opportunity trials is ongoing to also evaluate intratumoral cDCs upon treatment with Flt3L with or without CD40 agonist therapy (NCT04536077).

Nonetheless, the effect of Flt3L on the immune compartment has so far been mainly evaluated in non-tumor bearing mice and the impact of systemic Flt3L treatment on tumor DCs and tumor growth is still debated. Indeed, Salmon et al. have shown an increase in cDC1 and cDC2 in the tumor and an expansion of DC progenitors in bone marrow, blood and tumor upon Flt3L treatment in a B16 melanoma model (5). In addition, this resulted in a reduction in tumor growth. In contrast, upon systemic Flt3L treatment, Solheim et al. and Furumoto et al. did not identify changes in tumor DCs numbers, despite increases in DCs in the spleen (12, 24). In line with this, Flt3L treatment did not impact on tumor growth in respectively the cl-66 breast cancer model and the CT26 colorectal cancer model. In addition, Esche et al. observed an increase in DCs in the B16 melanoma and EL-4 lymphoma models, but not in the CL-8 melanoma model, while all three models showed a reduced tumor growth upon Flt3L treatment (25). This highlights the need for (1) an evaluation of tumor growth upon Flt3L treatment in different tumor models and (2) a more comprehensive analysis of cDC subsets in the tumor upon Flt3L treatment.

Therefore, in the current study, we have treated mice carrying 3 different tumor models (two orthotopic breast cancer models: E0771 and TS/A and the LLC lung cancer model) with Flt3L. In addition, in contrast to the previous studies, where Flt3L treatment was initiated either at tumor inoculation or at palpable tumor stage, we initiated Flt3L treatment when the tumor volume was approximately 100-200 mm^3^, a tumor volume more relevant for therapeutic interventions. Our results show is that in this context, systemic treatment with Flt3L does not reduce tumor growth in any of the tumor models tested. Therefore, the use of Flt3L as therapeutic agent in monotherapy will likely not provide any benefit for cancer patients.

One of the limitations of the above-mentioned studies evaluating DCs in the tumor is the lack of identification of different cDC subsets. Indeed, the studies described above do not take in account the full complexity of the cDCs within the TME with the presence of cDC1, cDC2, but also migDCs, a cDC-state by its expression of *Ccr7* and *Cd200* which has been attributed both antitumoral and immunoregulatory characteristics (7, 10, 26). In contrast to the lack of impact of Flt3L treatment on tumor growth, our results show that systemic Flt3L treatment increases both cDC1 and cDC2 in the tumor in all tumor models. Similarly to what has been shown in other models, cDC1s were in all models more responsive than cDC2s to Flt3L treatment (5). However, the response of pDCs to Flt3L treatment, which have been shown to also be induced upon Flt3L treatment (27), was only significantly increased in the E0771 and LLC tumor models and not the TS/A tumor model. Interestingly, while all cDC clusters increased upon Flt3L treatment, our attention was drawn to a distinct population of CD81^+^migcDC1s which was most prominently induced upon Flt3L treatment. A similar population, termed mDC1, has recently been identified in murine fibrosarcoma and was significantly reduced in Batf3 knock-out mice (28), corroborating our findings that CD81^+^migcDC1s originate from cDC1s. Indeed, also in flow cytometric analysis CD81^+^migcDC1s could be identified by their distinct expression of CD81 and XCR1 and were significantly increased upon Flt3L treatment in the E0771, TS/A and LLC tumor models. CD81 is a tetraspanin that was shown to be required for the formation of lamellipodia formation and DC migration and that is located at the immune synapse between T cells and DCs during their interaction (29, 30). Hence, CD81 could be involved in the interaction between CD81^+^migcDC1s and Tregs. While CD81^+^migcDC1s showed the strongest increase within the DC compartment upon Flt3L therapy, a specific targeting of these DCs, to unequivocally prove their regulatory role in the TME, is not feasible yet. Indeed, while CD81 is highly expressed on CD81^+^migcDC1s, it can also be expressed on cancer cells and other immune cells, such as Tregs and CD11b^+^GR1^+^ cells (31). Consequently, full body CD81 knock-out mice (31) or anti-CD81 antibodies (32) will not provide functional information on the role of CD81^+^migcDC1s.

Despite the increases in cDCs, CD8^+^ T-cells were not induced. Thereto, in the E0771 breast cancer model, we evaluated the T-cell compartment more in detail and observed a shift towards Tregs within the CD4^+^ T-cell population. Suggesting a potential immunosuppressive mechanism hampering the induction of an anti-tumor immune response.

Interestingly, our data show that both migDCs and CD81^+^migcDC1s derived from vehicle and Flt3L-treated animals had a Treg-inducing potential. However, due to their significant increases in the tumor upon Flt3L treatment, the shift towards Tregs could be mediated via the increase in CD81^+^migcDC1s and migDCs as has previously been shown in the context of autoimmunity (33, 34). This could in part be mediated via the increased CCL22 levels detected in the tumor supernatants upon Flt3L treatment. Indeed, it has previously been shown that tumor CCL22 can result in Treg attraction to the tumor and that CCL22 promotes DC-Treg interactions (9, 35). In addition, CITE-seq analysis also showed an increase in CCL22 expression in CD81^+^migcDC1s upon Flt3L treatment and could together with the CCL22 expression observed in migDCs be the cause of the increased CCL22 observed in the E0771 tumor supernatants. Indeed, CITE-seq analysis would suggest that CCL22 is only produced by migDCs and not by other DCs. However, the Treg-inducing potential of migDCs and CD81^+^migcDC1s could not be blocked by CCL22 inhibition. Nonetheless, while this excludes a function of CCL22 in the *in vitro* induction of Tregs by migDCs and CD81^+^migcDC1s, it does not exclude a potential function *in vivo* in the recruitment of Tregs to the tumor and suggests multiple mechanisms of action could be at play by which migDCs and CD81^+^migcDC1s induce Tregs in the tumor. Indeed, next to the alterations in CCL22, DCs could potentially also be immunoregulatory via other mechanisms such as for example the IL-2 dependent induction of Treg-proliferation by DCs, DC mediated production of IL-10, retinoic acid, IDO or TGFb (33, 36).

In addition, in E0771 TNBC tumors, we also observed high expression of PD-L1 in CD81^+^migcDC1, CD81^-^migcDC1 and migcDC2. To understand if an increase in these immunosuppressive mechanisms could be circumvented via an increased DC activation, Flt3L treatment was combined with CD40 agonist. Importantly, while Flt3L was shown to work synergistically with αCD40 in lung cancer (37), in E0771 breast cancer, Flt3L failed to improve the therapeutic response of αCD40 therapy. While we indeed showed increased cDC activation upon CD40 agonist treatment, we also observe a significantly increased expansion of CD81^+^migDC1s in mice treated with both Flt3L and CD40 agonist and this despite the early time point at which the tumors were evaluated. In addition, at the same time point, CCL22 levels were also significantly increased upon combination treatment coinciding with the increased presence of CD81^+^migDC1s. Furthermore, while Tregs at this time point are not yet increased upon Flt3L monotherapy, Tregs are significantly increased in E0771 tumors treated with the combination therapy in comparison to CD40 agonist-treated tumors.

To then understand if depleting Tregs could result in a synergistic effect in combination with Flt3L and CD40 agonist, E0771-tumor bearing mice were treated with Flt3L, CD40 agonist and anti-CD25 to deplete Tregs. Notably, Treg depletion and αCD40 therapy were synergistic, but the addition of Flt3L to this combination did not result in any added benefit. These results therefore suggest that the induction of Tregs is likely not the cause of the lack of anti-tumor immunity induced by the Flt3L-mediated increase in tumor cDCs. Alternatively, pDCs have also been shown to be suppressed by the TME and act to suppress anti-tumor immunity via Treg-activation (38). Therefore, these cells might also play a role in the immunosuppressive TME, nonetheless, the lack of increase in pDCs in the TS/A tumor model would suggest that pDCs are not the major contributing factor preventing an anti-tumor effect of Flt3L therapy and other mechanisms are also at play.

Overall, our data show that Flt3L treatment, while very effective in increasing intra-tumoral cDCs, is not able to induce anti-tumor immunity and reduce tumor growth. Therefore, while Flt3L treatment may be less promising as cancer therapy, it is still very attractive to increase the number of cDCs in the tumor, which could then be isolated for autologous cell-therapies (2).

## Materials and methods

### Mice

Female C57BL/6 and Balb/c mice were purchased from Janvier. All procedures followed the guidelines of the Belgian Council for Laboratory Animal Science and were approved by the Ethical Committee for Animal Experiments of the Vrije Universiteit Brussel (license 18-220-16, 19-220-33, 20-220-15, 20-220-17, 21-220-33, 23-220-03, 23-220-13, 23-220-21, 23-220-22).

### Tumor model

E0771 cells (ATCC) were cultured in DMEM (Gibco) supplemented with 10% (v/v) heat-inactivated fetal calf serum (FCS; Capricorn), 300 μg mL^−1^ L-glutamine (Gibco), 100 units mL^−1^ penicillin and 100 μg mL^−1^ streptomycin (Gibco). 5x10^5^ E0771 cells resuspended in 50 µL 50% (v/v) growth-factor reduced Matrigel (Corning)/HBSS (Gibco) were injected orthotopically in the fourth inguinal mammary fat pad. TS/A cells (provided by Dr. Vincenzo Bronte (Istituto Oncologico Veneto, Padova, Italy)) were cultured in supplemented (abovementioned) RPMI (Gibco). 10^6^ TS/A cells resuspended in 50 µL HBSS were injected orthotopically in the fourth inguinal mammary fat pad of Balb/c mice. LLC cells (ATCC) were cultured in supplemented (abovementioned) DMEM. 10^6^ LLC cells resuspended in 100 µL HBSS were injected subcutaneously in the right flank of C57BL/6 mice. Tumor volumes were monitored with caliper measurements and calculated using the formula: V = π × (d^2^ × D)/6, where D is the longest diameter and d the shortest diameter.

### Treatments

After randomization based on tumor volume, mice were injected intraperitoneally (ip) with 30 µg recombinant Flt3L (BioXCell - BE0098) or vehicle (HBSS) daily for 12, 9, or 6 days till sacrifice for E0771, 9 days till sacrifice for TS/A and 7 days till sacrificed for LLC. For CD40 agonist treatments, a single dose of 100 μg of CD40 (clone: FGK4.5; BioXCell) agonist antibody or rat IgG2a isotype control (clone 2A3; BioXCell) was administered ip. For Treg-depletions, a single dose of 20 μg of αCD25 (ONCC4, kindly provided by Oncurious) or mouse IgG2a isotype control (clone C1.18.4; BioXCell) was administered ip.

### Tissue dissociation

Tumors, tumor-draining lymph nodes, spleens and bone marrow were collected as previously described (2, 10).

### Flow cytometry and cell sorting

Cells were labeled for flow cytometry analysis as previously described (10) and labeled with the antibodies listed in **Supplementary Table 1**. Flow cytometry was performed on BD FACSymphony A3 or BD FACSCanto.

Prior to DC sorting, tumor single cell suspensions were loaded in a Lymphoprep™ gradient to enrich mononuclear lymphocytes. After incubation with rat anti-mouse CD16/CD32 in FACS buffer for 10 min on ice and incubation with PerCP-Cy5.5 – CD11b, PE – CD81, PE Texas Red – CD64, PeCy7 – CCR7, FITC – MHCII, APC-Cy7 – CD11c and APC – XCR1 diluted in FACS buffer for 20 min at 4°C. Naive CD4^+^ T cells were sorted from splenocytes after incubation with rat anti-mouse CD16/CD32 in FACS buffer for 10 min on ice and incubation with PerCP-Cy5.5 – CD62L, PE – CD4, FITC – CD44 and APC – TCRb diluted in FACS buffer for 20 min at 4°C. Cells were sorted with a BD FACSAria™II or with a Cytek Aurora CS.

### Coculture cDCs and naive CD4 T-cells

10^5^ splenic CD44^-^CD62L^+^CD4^+^ T cells from naive mice were cultured with either 3000 CD81^+^migcDC1s or 3000 CD81^-^migDCs (CD81^-^migcDC1s and migcDC2s) from Flt3L-treated or vehicle-treated tumors for 5 days in RPMI 1640 (Gibco) supplemented with 10% (v/v) heat-inactivated FCS (Gibco), 300 μg mL^−1^ L-glutamine (Gibco), 100 units mL^−1^ penicillin, 100 μg mL^−1^ streptomycin (Gibco), 1 mM non-essential amino acids (Invitrogen), 1 mM sodium pyruvate (Invitrogen) and 0.02 mM 2-mercapto ethanol (Invitrogen). T-cell differentiation was initiated in respective wells with 5 ng ml^-1^ murine IL-2 (Biolegend) and 1 µg mL^-1^ anti-CD3 (clone 145-2C11, BD Biosciences) on day 2 and day 4. To inhibit CCL22, anti-mouse CCL22/MDC polyclonal antibody (2 μg mL^−1^) (R&D systems) was administered to the respective wells.

### Single cell RNA-seq/CITE-seq

Tumor-bearing mice were treated at day 12 after E0771 inoculation and treated for 9 consecutive days with vehicle or Flt3L. Similarly sized tumors collected at day 21 after tumor inoculation were pooled from three mice/condition. The regular tissue processing procedure was followed, with the addition of actinomycin D (ActD, Sigma-Aldrich, A1410-5MG) to each buffer. Tumor collection was performed in 30 µM ActD, enzyme incubation and subsequent filtering in 15 µM ActD, and all other steps in 3 µM ActD. 10^6^ cells were stained with anti-CD45 APC-Cy7 (1:50, Biolegend), a mouse cell surface protein antibody panel containing 167 oligo-conjugated antibodies for CITE-seq and TruStain FcX™ PLUS Antibody (1:25; Biolegend) in Phosphate buffered saline (PBS) with 0.04% bovine serum albumin (BSA) for 30 minutes on ice. After incubation, cells were incubated with 7AAD (1:100) 5 minutes prior to sorting. 8x10^4^ living CD45^+^ cells were sorted using BD FACSAriaTM II and collected in 500 μl PBS + 0.04% BSA and 3 μM ActD. The 10× genomics single-cell bead-in emulsions and CITE-seq libraries were prepared as described previously (39). The mean RNA reads per cell for the vehicle and Flt3L CITE-seq data were 28036 and 21833 with an RNA sequencing saturation metric of 43.7% and 45% respectively. The ADT libraries yielded 4033 and 3907 mean reads per cell, and 69.2% and 72.7% ADT sequencing saturation, for the vehicle and Flt3L conditions respectively. For filtering the low-quality cell barcodes, associated with empty droplets, the “emptyDrops” function of the DropletUtils package (v.1.10.2) has been applied on the RNA expression data, using an FDR cutoff of 0.01. The gene expression matrices were further filtered for outliers for mitochondrial genes percentage and low-abundance genes as described previously, using the Scater package (v.1.18.3) (40). Library size normalization and unsupervised Leiden clustering were performed with Seurat v.3.2.3. The obtained clustering was visualized via Uniform Manifold Approximation and Projection (UMAP) plots. Differential expression analysis was done using Wilcoxon Rank Sum test to identify genes, specific for each cluster. Bonferroni correction has been applied for adjustment of the P values. The processing of the ADT expression matrix was done as described previously (40).

### Single cell RNA velocity analysis

The estimation of the RNA velocity was performed with the velocyto python package v.0.17.16 and the R package velocyto.R_0.6. The spliced/unspliced expression matrices were generated with velocyto with using the option for masking the repetitive elements. The expressed repeat annotations for mm10 were downloaded from the UCSC genome browser. The genes of the resulting expression matrices were filtered based on the minimum average expression in at least one of the clusters (0.1 and 0.05 counts for the spliced and unspliced matrix, respectively). Then, RNA velocity was estimated with the velocyto.R_0.6 package using gene-relative model with k=20 cell kNN pooling and using top/bottom 2% quantiles for gamma fit (“fit.quantile” option). The RNA velocities were visualized on the UMAP embeddings via correlation-based transition probability matrix within the kNN graph using default parameters.

### Gene ontology enrichment

Gene ontology enrichment analysis was performed using the Metascape (http://metascape.org/) online tool with default parameters (41).

### Software

Flow cytometry data was analyzed using FlowJo™. CITE-seq data was analyzed and graphs were generated using R version 4.0.3, DropletUtils version 1.10.2, scater version 1.18.3, Seurat version 3.2.3, Nichenet version 1.1.0, velocyto version 0.17.16 and velocyto.R_0.6. All schematic figures were created using BioRender.com.

### Statistics

Statistics and graphs were generated using GraphPad Prism. All graphs represent the mean ± standard error of the mean (SEM Statistics and graphs were generated using GraphPad Prism. All graphs represent the mean ± standard error of the mean (SEM). For data showing the expression of cell types as percentage of the main population, a LOGIT transformation was performed to correct for the non-normal distribution and/or the heteroscedasticity of the data. Consequently, statistical analysis using unpaired t-tests (**Figures 1B**, **2F**, **4C**, **S3F, S4C–F, S5E, S6A**.), one-way analysis of variance (ANOVA) (**Figures 3E**, **4B**, **S5B–D, H, S6B–E**) or two-way ANOVA were performed (**Figures 1A**, **S1C–F, S4A, B**). Both one-way and two-way ANOVA were followed by a *post hoc* analysis and a Bonferroni correction for multiple testing. Tumor growth curves were compared by mixed-effects two-way ANOVA with multiple comparisons tests. Survival curves were subjected to Kaplan-Meier survival analyses. For statistically significant differences, the p value is indicated in graphs as the following: *p<0.05, **p<0.01, ***p<0.001, ****p<0.0001.

## Data availability statement

The data associated with this study are available in the main text or the supplementary materials. ScRNA-seq raw data are deposited in the GEO (NCBI) repository, accession number GSE225539.

## Ethics statement

The animal study was reviewed and approved by Ethical Committee for Animal Experiments of the Vrije Universiteit Brussel.

## Author contributions

EC, SD, and DLao designed the experiments. EC, JB, AD, PB, YE, DLac, MŽ, AH, and SD performed the experiments. EC, DK, and SD performed analyses on the experimental data. EC and DK performed bioinformatics analyses. EC, DK, SD, and DLao wrote the manuscript. AH and JVG gave advice on the experimental design and manuscript. SD and DLao supervised the study. All authors contributed to the article and approved the submitted version.
