# Supplementary figures and images for: Flt3L therapy increases the abundance of Treg-promoting CCR7+ cDCs in preclinical cancer models
Source: Front Immunol. 2023 Aug 9;14:1166180. doi: 10.3389/fimmu.2023.1166180 (PMC10445485; doi:10.3389/fimmu.2023.1166180)

Supplementary Figure 1

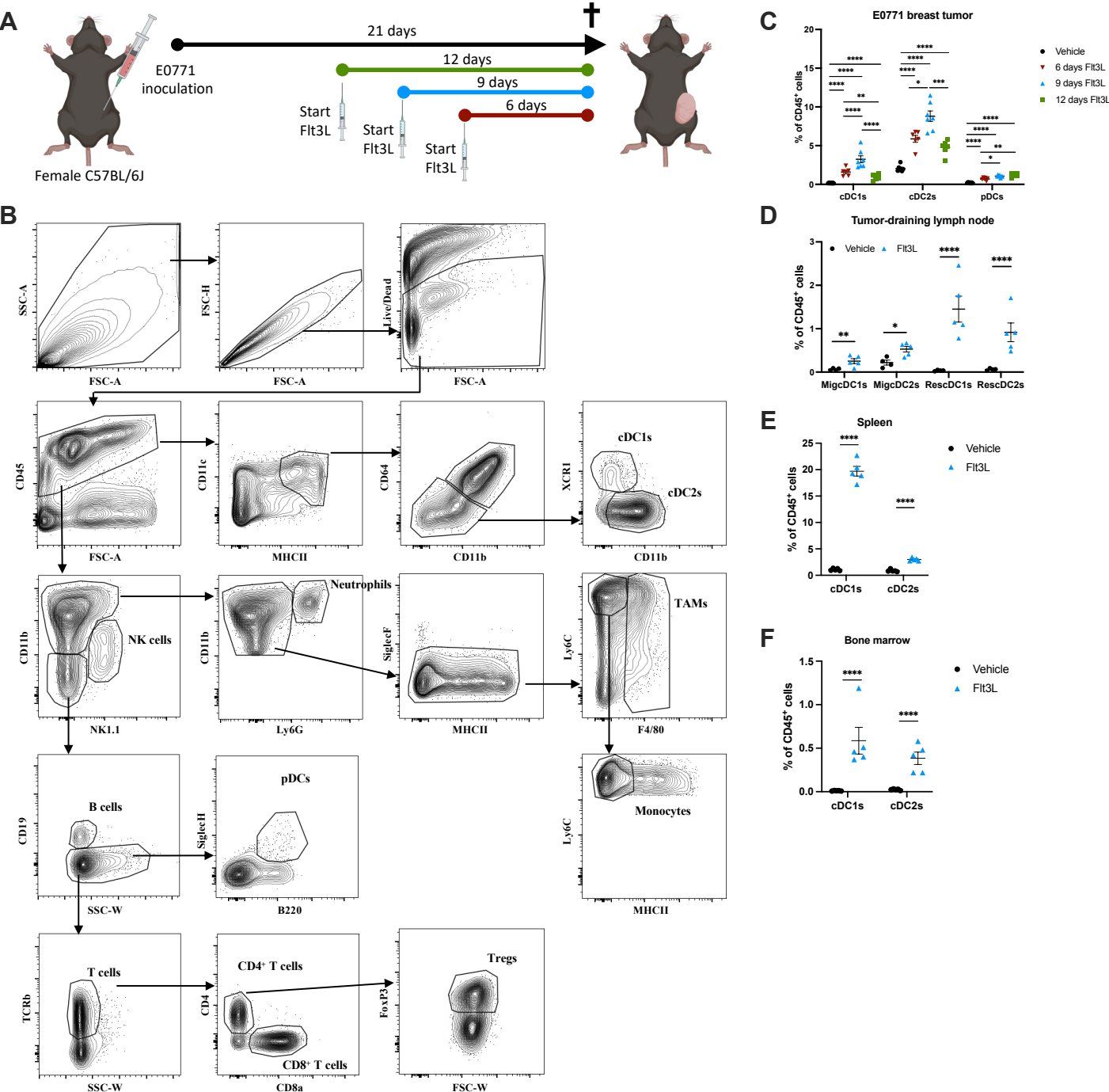

Supplement: Supplementary file 1 [file Image_1.pdf]

# Supplementary Figure 3

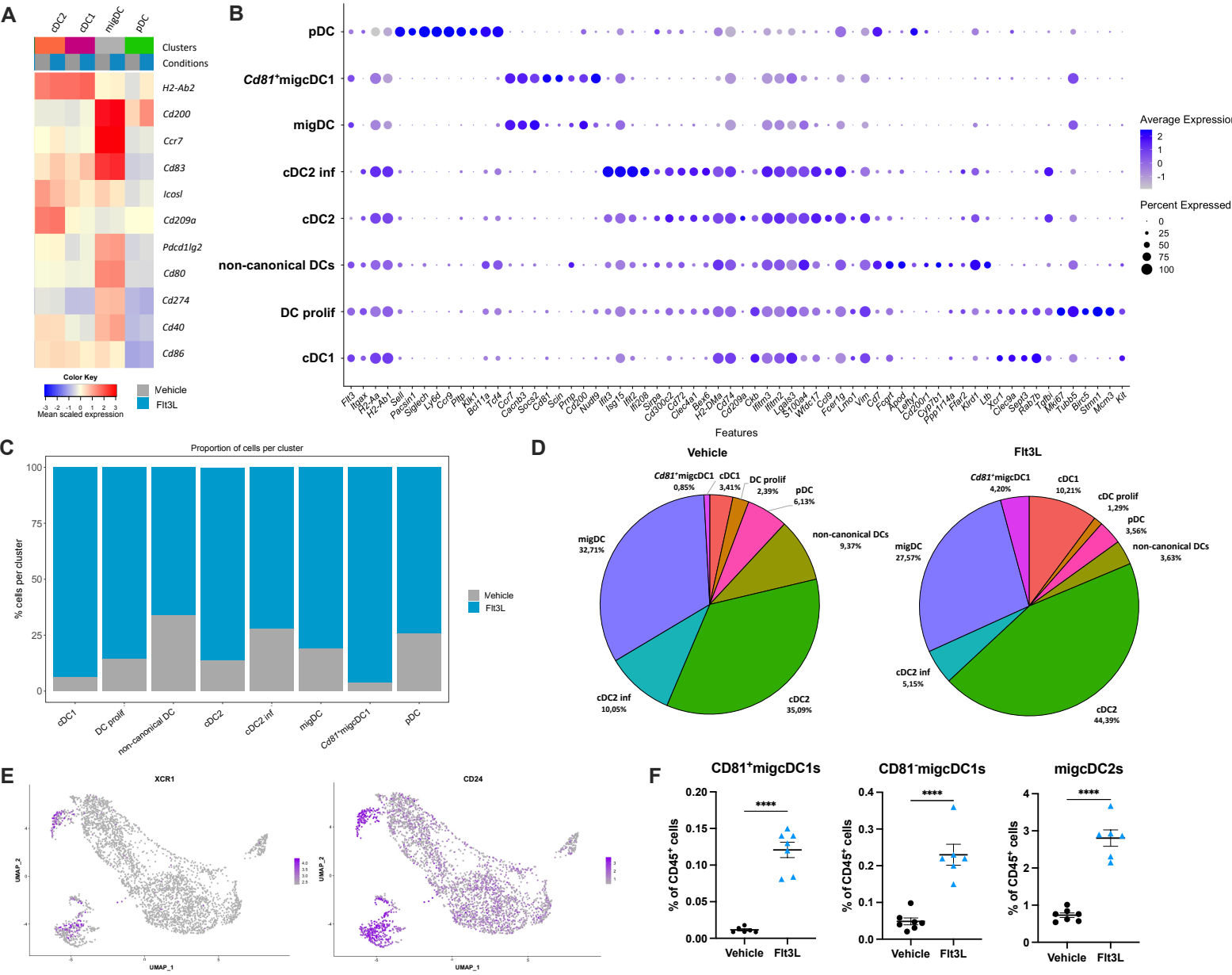

Supplement: Supplementary file 3 [file Image_3.pdf]

### Supplementary Figure 4

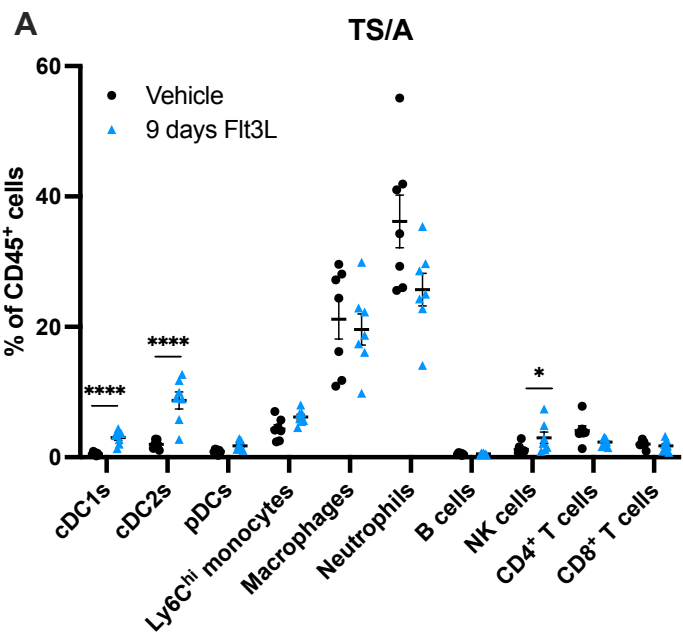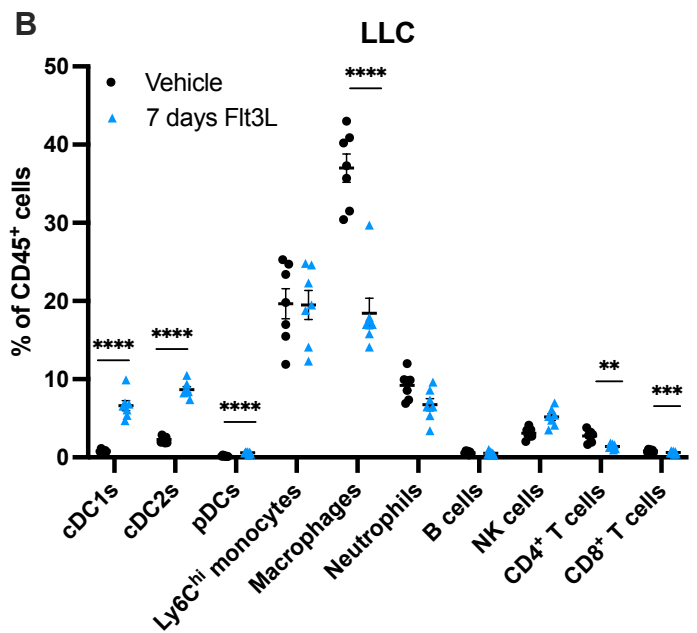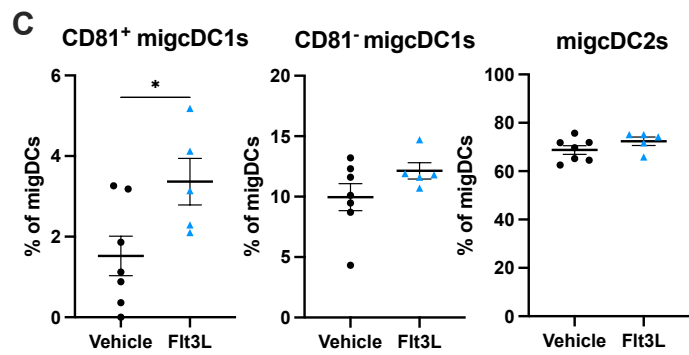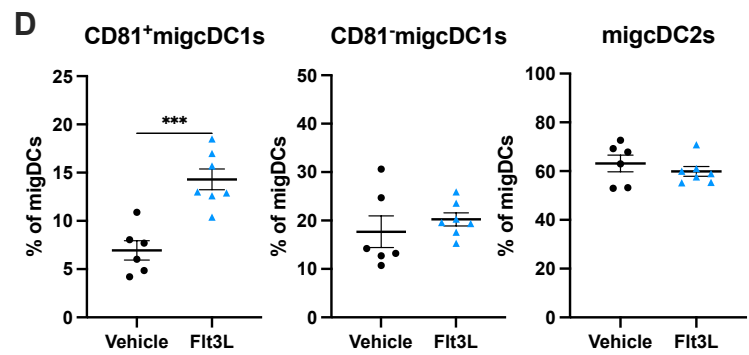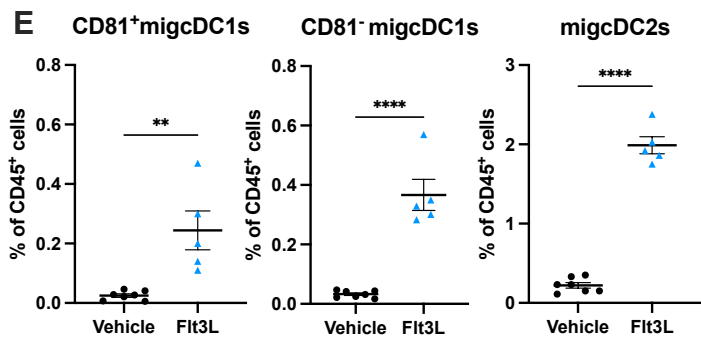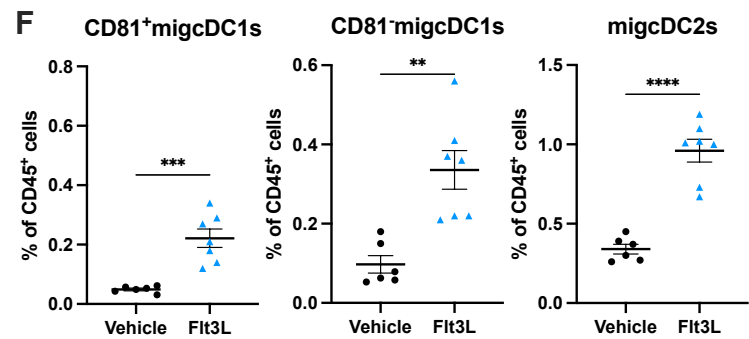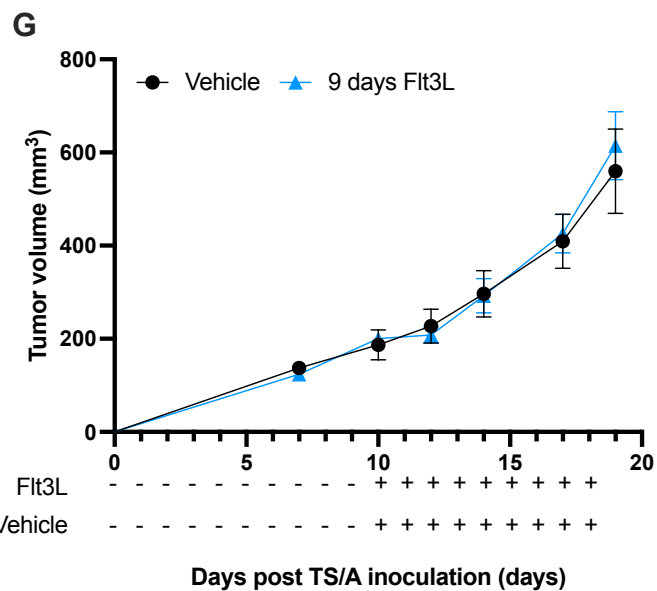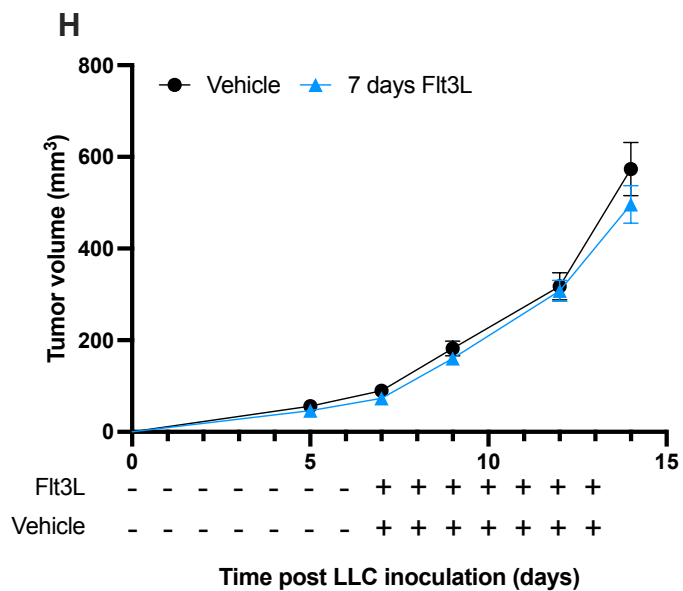

Supplement: Supplementary file 4 [file Image_4.pdf]

# Supplementary Figure 6

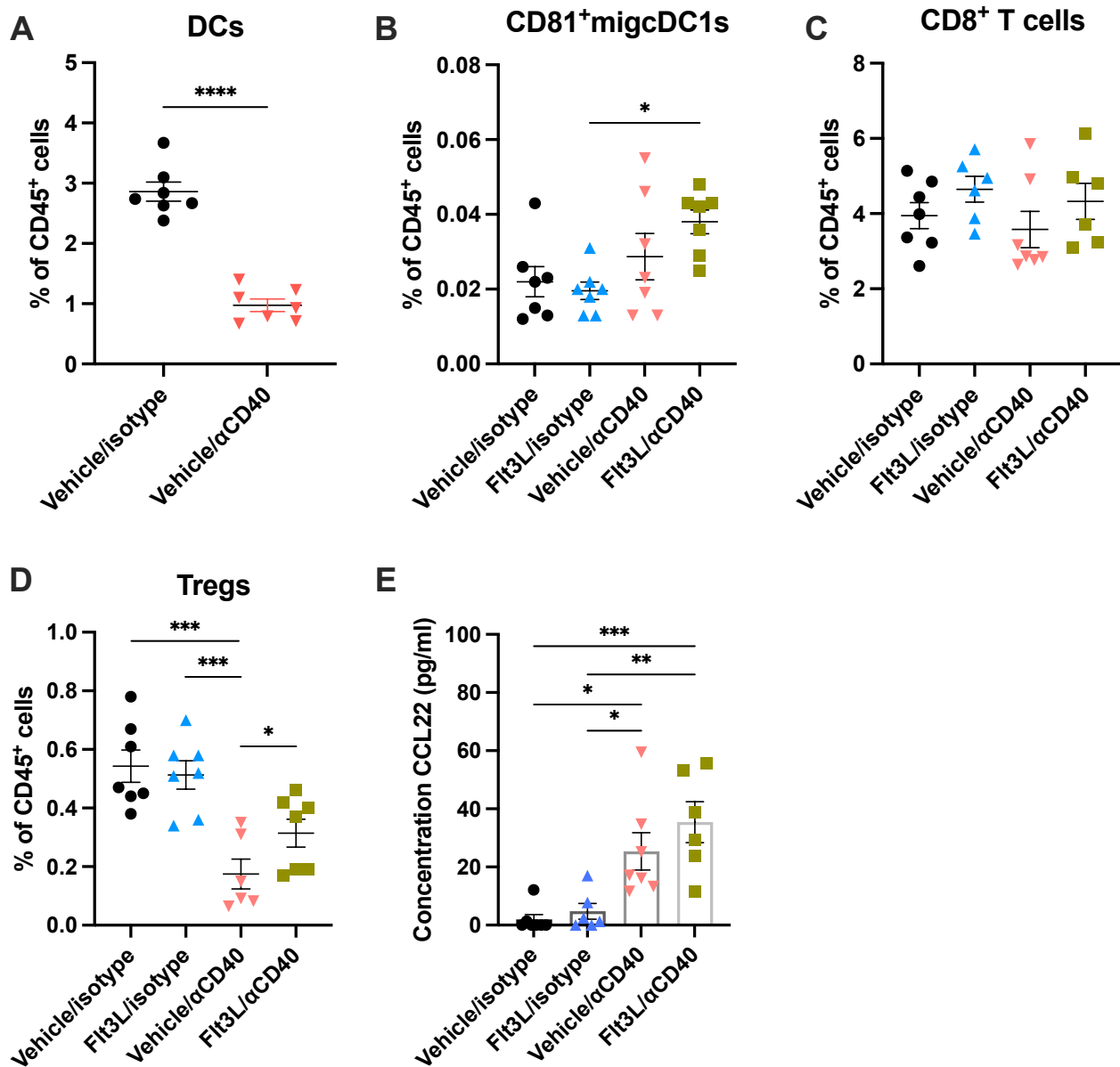

Supplement: Supplementary file 6 [file Image_6.pdf]
