# Supplementary material for: Flt3L therapy increases the abundance of Treg-promoting CCR7+ cDCs in preclinical cancer models
Source: Front Immunol. 2023 Aug 9;14:1166180. doi: 10.3389/fimmu.2023.1166180 (PMC10445485; doi:10.3389/fimmu.2023.1166180)

Supplementary Figure 5

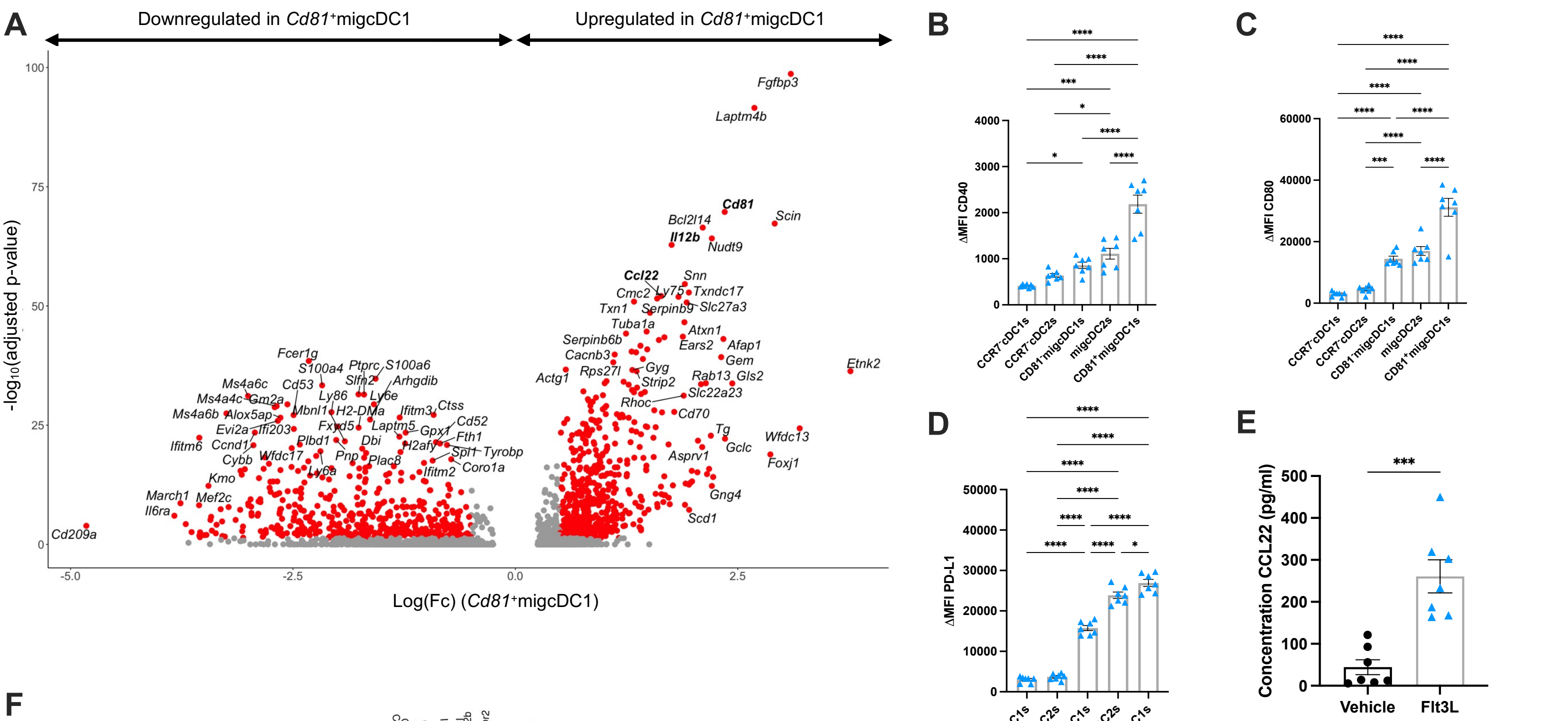

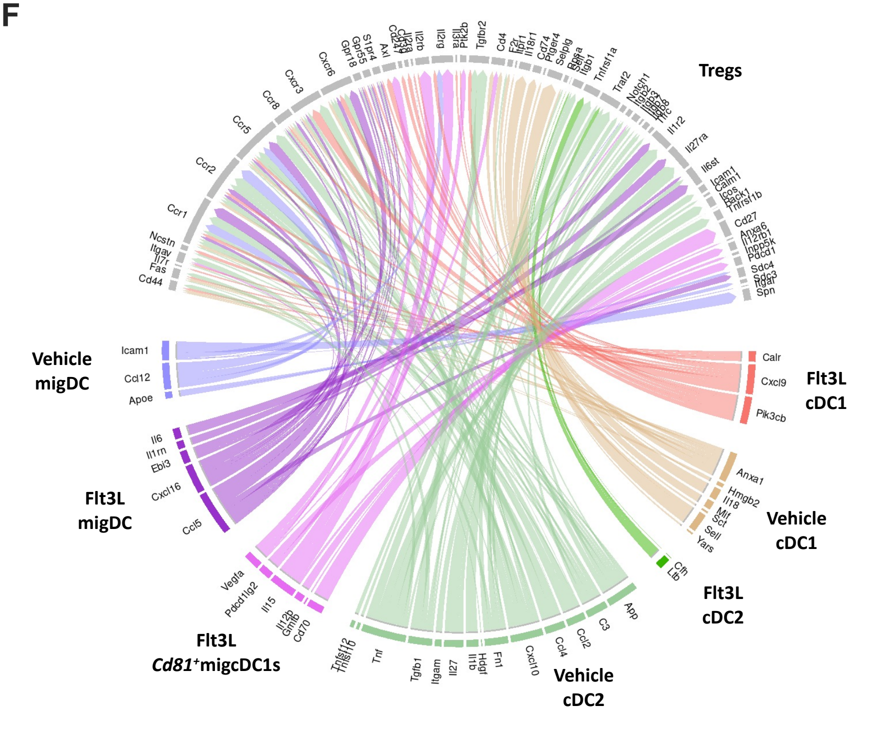

G

sender	receiver	auroc	aupr	pearson	frac_pos_predicted	frac_neg_predicted	fisher.p.val
Flt3L cDC1	Tregs	0.501	0.066	0.007	0.056	0.050	0.376
Vehicle cDC1	Tregs	0.522	0.073	0.031	0.075	0.048	0.083
Flt3L cDC2	Tregs	0.519	0.067	0.010	0.052	0.050	0.478
Vehicle cDC2	Tregs	0.525	0.073	0.033	0.076	0.049	**0.076**
Flt3L Cd81⁺migcDC1s	Tregs	0.538	0.083	0.054	0.089	0.048	**0.012**
Flt3L migDC	Tregs	0.486	0.060	-0.016	0.043	0.051	0.746
Vehicle migDC	Tregs	0.484	0.065	0.003	0.053	0.050	0.465

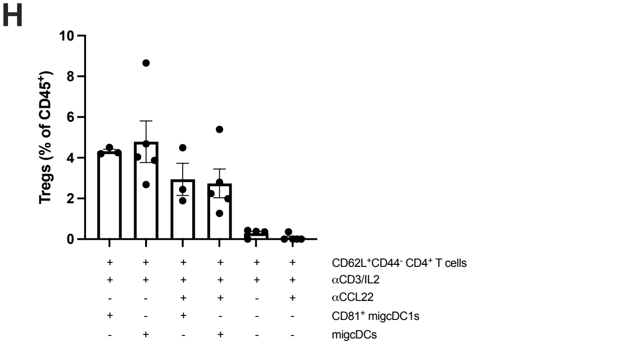

Supplement: Supplementary file 5 [file Image_5.pdf]
