# Supplementary material for: Flt3L therapy increases the abundance of Treg-promoting CCR7+ cDCs in preclinical cancer models
Source: Front Immunol. 2023 Aug 9;14:1166180. doi: 10.3389/fimmu.2023.1166180 (PMC10445485; doi:10.3389/fimmu.2023.1166180)
Supplement: Supplementary file 7 [file DataSheet_1.docx]

**Supplemental materials and figures**

**SUPPLEMENTAL MATERIALS AND METHODS**

**CCL22 ELISA**

The CCL22 concentration in tumor cell suspension supernatants was measured via a sandwich ELISA according to the manufacturer’s instructions (R&D Systems, DY439). Optical density measurements were obtained using a Varioskan™ LUX Multimode Microplate Reader (Thermo Scientific™) set to 450 and 540 nm, whereby the latter were used for background correction.

**Cell-cell communication modelling**

Ligand-receptor interactions between the different cDC/migDC subsets and the Tregs were predicted via the NicheNet algorithm (Nichenetr, v1.1.0). Nichenet uses a prior model of intercellular interactions, based on ligand-receptor, signaling and gene regulatory data sources, which were aggregated into an integrated network and used to calculate regulatory potential score for each ligand-target link. NicheNet ranks the ligands expressed in the sender cells and estimates a ligand activity score, based on how well the target genes of the ligands from the prior model predict the observed gene set of interest compared to a background set of genes. The gene set of interest refers to the genes in the receiver population that are potentially affected by the ligands of the sender cells. As ligand activity metric, Pearson correlation coefficient between the prior regulatory potential scores and the gene set of interest assignments is used, so higher score indicates better predictive ligands. Then, Nichenet infers the potential receptors (expressed in the receiver cells) and target genes (belonging to the gene set of interest and to the 250 most strongly predicted targets from the prior model of at least one of the selected ligands) of the top ranked ligands.

The cDC1, cDC2 and migDC populations were defined as sender cells, and the Tregs – as receiver cells. The gene set of interest was defined as the differentially expressed genes between the FLT3L and naive condition in the Tregs (p_val <0.1). We have selected the top 40 ligands by ligand activity for further analysis. Next, we wanted to evaluate which DC subset shows strongest evidence of ongoing intercellular communication with the Treg population. We assigned each of the 40 prioritized ligands to the DC subset, where it was expressed highest. Then, we assessed how well the prioritized ligands of each DC subset can predict the gene set of interest, based on a multi-ligand random forest model with cross-validation. The classification model returns a probability for each target gene if it belongs to the gene set of interest or not. Then, we applied a Fisher’s exact test to test if the gene set of interest genes are enriched in the top-predicted target genes list. A high fraction of the gene set of interest genes in the prior model target genes for the ligands suggests that actual cell-cell communication is ongoing between the sender and receiver cells.

**SUPPLEMENTAL FIGURES AND TABLES**

**Supplemental Figure 1.** Flt3L treatment significantly increases dendritic cells in the E0771 tumor microenvironment. **A.** Graphical representation of the experimental design where E0771 tumor bearing mice are treated for 6, 9 or 21 consecutive days with vehicle or Flt3L. **B,** Gating strategy for flow cytometry to identify different cell populations within the E0771 tumor microenvironment. **C,** Percentage of cDC1, cDC2 and pDC in the CD45^+^ cell population of E0771 tumors as described in A. Data from 2 independent experiments (n=7). **D,** Percentage of Migratory cDC1 (MigcDC1), MigcDC2, Resident cDC1 (RescDC1) and RescDC2 in CD45^+^ cell population of tumor draining lymph nodes after 9 days of vehicle or Flt3L treatment. Data from 2 independent experiments (n=4-5). **E,F,** Percentage of cDC1 and cDC2 in bone marrow (E) and spleen (F) after 9 days of vehicle or Flt3L treatment. Data from 2 independent experiments (n=4-5). *, *P < 0.05*; **, *P < 0.01*; ***, *P < 0.001*; ****, *P < 0.0001*

**Supplemental Figure 2.** Flt3L treatment shifts the T-cell compartment towards regulatory T cells. **A,** Dotplot showing 19 cellular clusters of CITE-seq analysis performed on the CD45^+^ fraction of E0771 tumors collected from 9 days vehicle and Flt3L-treated animals. Clusters were annotated based on the expression of canonical marker genes. Data from 1 experiment (n=3). **B,** Split UMAP of CITE-seq analysis performed on the CD45^+^ fraction of E0771 tumors collected from 9 days vehicle and Flt3L-treated animals. **C,** UMAP of the re-analyzed T-cell compartment of CITE-seq analysis performed on the CD45^+^ fraction of E0771 tumors collected from 9 days vehicle and Flt3L-treated animals. **D,** Dotplot showing 6 cellular clusters obtained after re-analysis of the T-cell compartment of CITE-seq analysis performed on the CD45^+^ fraction of E0771 tumors collected from 9 days vehicle and Flt3L-treated animals. Clusters were annotated based on the expression of canonical marker genes. **E,** Violin plots showing expression of *Foxp3*, *Lag3*, *Klrg1*, *Ikzf2*, *Tnfrsf4*, *Ccr8*, *Il2ra*, *Cd44*, *Cd69* and *Ctla4* on regulatory T cells (Tregs) identified in the CITE-seq analysis performed on the CD45^+^ fraction of E0771 tumors collected from 9 days vehicle and Flt3L-treated animals. **F,** Heatmap representing average expression of *Tcf7*, *Sell*, *Gzmb*, *Cd69*, *Ifng*, *Pdcd1*, *Cd44*, *Il2ra*, *Tox* and *Lag3* in T-cell clusters identified after re-clustering of the CITE-seq analysis performed on the CD45^+^ fraction of E0771 tumors collected from 9 days vehicle and Flt3L-treated animals.

**Supplemental Figure 3.** Flt3L therapy induces a CD81^+^ CCR7^+^ cDC1 activation status. **A,** Heatmap representing average expression of *H2-Ab2*, *Cd200*, *Ccr7*, *Cd83*, *Icosl*, *Cd209a*, *Pdcd1lg2*, *Cd80*, *Cd274*, *Cd40* and *Cd86* in DCs in CITE-seq analysis performed on the CD45^+^ fraction of E0771 tumors collected from 9 days vehicle and Flt3L-treated animals. **B,** Dotplot with 8 cellular clusters obtained after re-analysis of the DC compartment of CITE-seq analysis performed on the CD45^+^ fraction of E0771 tumors collected from 9 days vehicle and Flt3L-treated animals. Clusters were annotated based on the expression of canonical marker genes. **C,** Bar graph showing the relative contribution of each condition in the DC clusters identified in the DC compartment of CITE-seq analysis performed on the CD45^+^ fraction of E0771 tumors collected from 9 days vehicle and Flt3L-treated animals. **D,** Pie charts of the different DC clusters identified in the DC compartment of CITE-seq analysis performed on the CD45^+^ fraction of E0771 tumors collected from 9 days vehicle and Flt3L-treated animals. **E,** Feature plot showing the protein expression of XCR1 and CD24 in the DC clusters of CITE-seq analysis performed on the CD45^+^ fraction of E0771 tumor collected from 9 days vehicle and Flt3L-treated animals. **F,** Flow cytometric analysis of CD81^+^migcDC1, CD81^-^migcDC1 and migcDC2 as percentage of CD45^+^ cells from E0771 tumors collected after 9 days of vehicle or Flt3L treatment. Data from 2 independent experiments (n = 6-7). ****, *P < 0.0001*

**Supplemental Figure 4.** Flt3L therapy increased the presence of CD81^+^migcDC1s in TS/A and LLC. **A,** Frequency of immune populations within TS/A tumors treated for 9 days with vehicle or Flt3L (n=7). **B,** Frequency of immune populations within LLC tumors treated for 7 days with vehicle or Flt3L (n=7). **C,** Percentages of CD81^+^migcDC1, CD81^-^migcDC1 and migcDC2 within migDCs in TS/A tumors (n=5-7). **D,** Percentages of CD81^+^migcDC1, CD81^-^migcDC1 and migcDC2 within migDCs in LLC tumors (n=7). **E,** Percentages of CD81^+^migcDC1, CD81^-^migcDC1 and migcDC2 within CD45^+^ cells in TS/A tumors (n=5-7). **F,** Percentages of CD81^+^migcDC1, CD81^-^migcDC1 and migcDC2 within CD45^+^ cells in LLC tumors (n=7). **G,** TS/A tumor growth upon 9 days of vehicle or FLt3L treatment. **H,** LLC tumor growth upon 7 days of vehicle or FLt3L treatment (n=7). Representative data from 2 experiments. *, P < 0.05; **, P < 0.01, ***, P < 0.001; ****, P < 0.0001.

**Supplemental Figure 5.** Flt3L-induced tumor-associated CD81^+^migcDC1 are potent Treg inducers. **A,** Volcano plot showing genes up and downregulated in *Cd81*^+^migcDC1 relative to the other DC clusters identified in the DC compartment in the CITE-seq analysis of CD45^+^ cells isolated from E0771 tumors of 9-day Flt3L treated mice with red dots representing significantly upregulated genes. **B-D,** Delta mean fluorescence intensity (∆MFI) of CD40 (B), CD80 (C) and PD-L1 (D) in the different dendritic cell subsets in the E0771 tumor microenvironment of 9-day vehicle or Flt3L treated mice. Data from 2 independent experiments (n=7). **E,** CCL22 levels detected in E0771 tumor supernatants collected after 9 days of vehicle or Flt3L treatment. Data from 1 experiment (n=7). **F,** Circle plot showing links between top predicted ligands in the DC subsets and their receptors found in Tregs identified from the CITE-seq analysis of CD45^+^ cells isolated from E0771 tumors of 9-day Flt3L treated mice. The width of the arrows corresponds to the regulatory potential based on prior knowledge for interaction. **G,** Assessment of the potential of the prioritized ligands of each DC subset to predict the gene set of interest. The prediction is performed with a multi-ligand random forest model with cross-validation. Auroc - area under the receiver operating characteristic; aupr – area under the precision-recall curve; pearson - Pearson's coefficient; frac_pos_predicted - percentage of gene set of interest genes within the top 5% predicted targets of the ligands; frac_neg_predicted - percentage of non-gene set of interest genes within the top 5% predicted targets of the ligands; fisher.p.val - Fisher’s p-value evaluating if the gene set of interest genes are enriched in the top-predicted target genes list. **H,** Percentage of Tregs within CD45^+^ cells after coculture of sorted CD81^+^migcDC1 and migDCs (CD81^-^migcDC1 and migcDC2) with naive CD44^-^CD62L^+^CD4^+^ T cells in the presence or absence of a polyclonal anti-CCL22 antibody (2 µg/mL). Representative data from 2 experiments (n=5). *, P < 0.05; **, P < 0.01; ***, P < 0.001; ****, P < 0.0001

**Supplemental Figure 6.** Flt3L therapy does not improve the αCD40-mediated reduction of tumor growth. **A,** DCs as percentage of CD45^+^ cells within the E0771 tumor 2 days after isotype or αCD40 treatment as depicted in Figure 4A. Data from 2 independent experiments (n=7). **B,** Percentage of CD81^+^migcDC1 within DCs of E0771 tumors collected respectively 3 days and 2 days after treatment start of vehicle/Flt3L or isotype/αCD40 as depicted in Figure 4A. Data from 1 experiment (n=5-7). **C,D,** CD8^+^ T cells (C) and regulatory T cells (Tregs; D) as percentage of CD45^+^ cells within E0771 tumors collected respectively 3 days and 2 days after treatment start of vehicle/Flt3L or isotype/αCD40 as depicted in Figure 3C. Data from 1 experiment (n=5-7). **E,** Concentration of CCL22 detected in the supernatants of E0771 tumors collected respectively 3 days and 2 days after treatment start of vehicle/Flt3L or isotype/αCD40 as depicted in Figure 4A. Data from 1 experiment (n=6-7). *, P < 0.05; **, P < 0.01; ***, P < 0.001; ****, P < 0.0001

**Supplemental table S1**

| Target | Clone |
| --- | --- |
| Armenian HamsterIgG1 | HTK888 |
| B220 | RA3-6B2 |
| CCR7 | 4B12 |
| CCR8 | REA921 |
| CD3 | 17A2 |
| CD3e | 145-2c11 |
| CD4 | RM4-5 |
| CD4 | GK1.5 |
| CD8a | 53-6.7 |
| CD11b | M1/70 |
| CD11c | N418 |
| CD11c | HL3 |
| CD19 | MB19-1 |
| CD19 | 1D3 |
| CD24 | M1/69 |
| CD40 | 3/23 |
| CD45 | 30-F11 |
| CD64 | X54-5/7.1 |
| CD80 | 16-10A1 |
| CD81 | Eat2 |
| CD274 | MIH5 |
| F4/80 | CI:A3-1 |
| F4/80 | T45-2342 |
| FOXP3 | MF23 |
| FOXP3 | FJK-16s |
| Ly6C | AL-21 |
| Ly6G | 1A8 |
| MHCII | M5/114.15.2 |
| MHCII | 2G9 |
| NK1.1 | PK136 |
| RatIgG2a, k | eBR2a |
| RatIgG2a, k | RTK2758 |
| Sca-1 | D7 |
| SiglecF | S17007L |
| SiglecF | E50-2440 |
| SiglecH | 551 |
| SiglecH | 440c |
| TCRb | H57-597 |
| XCR1 | ZET |
