# Supplementary material for: Flt3L therapy increases the abundance of Treg-promoting CCR7+ cDCs in preclinical cancer models
Source: Front Immunol. 2023 Aug 9;14:1166180. doi: 10.3389/fimmu.2023.1166180 (PMC10445485; doi:10.3389/fimmu.2023.1166180)

Supplementary Figure 2

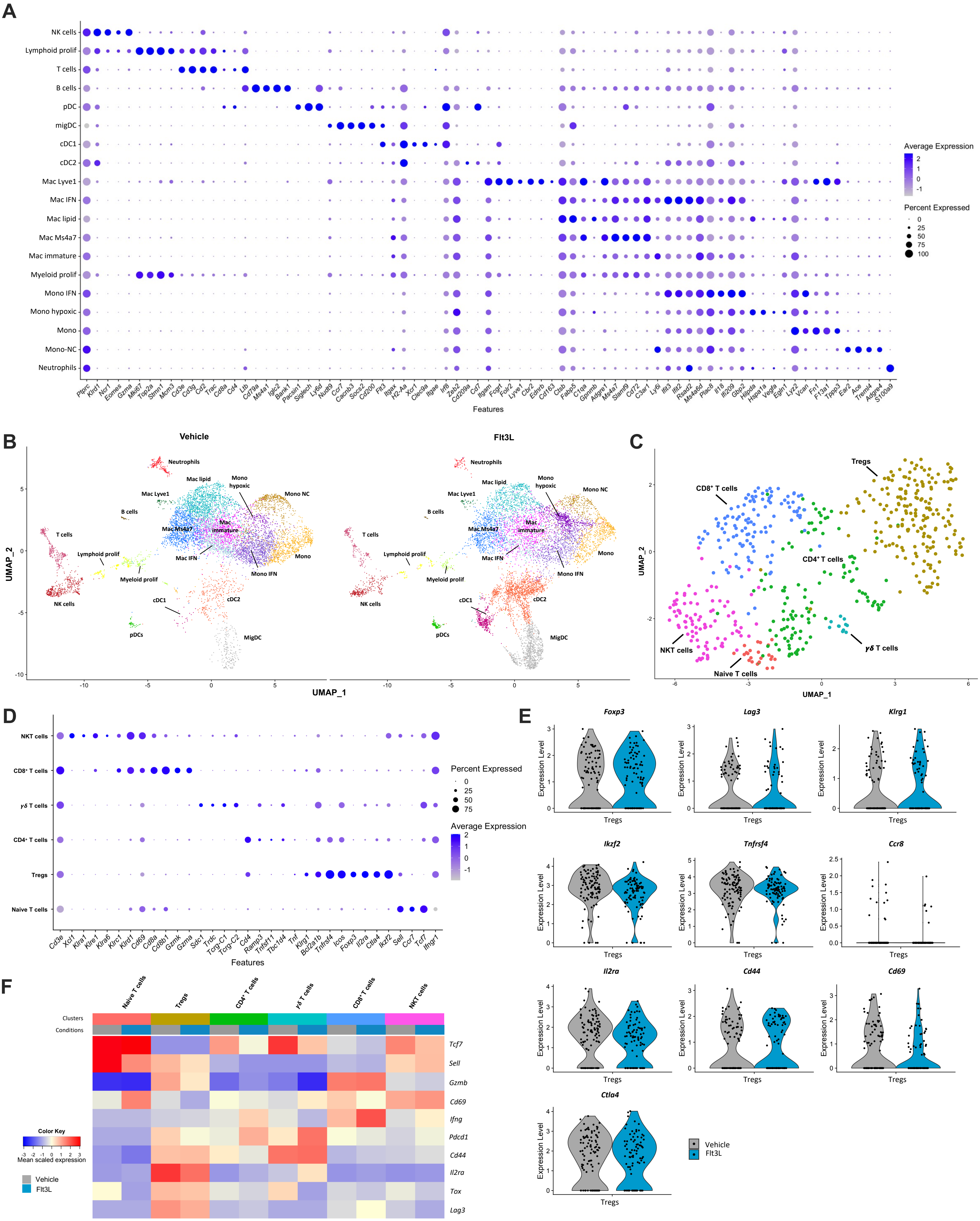

Supplement: Supplementary file 2 [file Image_2.pdf]
